# Recognition of activities of daily living in healthy subjects using two ad-hoc classifiers

**DOI:** 10.1186/s12938-015-0050-4

**Published:** 2015-06-06

**Authors:** Prabitha Urwyler, Luca Rampa, Reto Stucki, Marcel Büchler, René Müri, Urs P Mosimann, Tobias Nef

**Affiliations:** Gerontechnology and Rehabilitation Group, University of Bern, Bern, Switzerland; University Hospital of Old Age Psychiatry, University of Bern, Bern, Switzerland; Perception and Eye Movement Laboratory, Departments of Neurology and Clinical Research, University Hospital Inselspital, University of Bern, Bern, Switzerland; ARTORG Center for Biomedical Engineering Research, University of Bern, Bern, Switzerland

**Keywords:** Naïve Bayes, Random Forest, Activities of daily living, ADL recognition, Wireless sensor, Forward chaining inference engine, Circadian activity rhythm, Rule based inference, Classifiers

## Abstract

**Background:**

Activities of daily living (ADL) are important for quality of life. They are indicators of cognitive health status and their assessment is a measure of independence in everyday living. ADL are difficult to reliably assess using questionnaires due to self-reporting biases. Various sensor-based (wearable, in-home, intrusive) systems have been proposed to successfully recognize and quantify ADL without relying on self-reporting. New classifiers required to classify sensor data are on the rise. We propose two ad-hoc classifiers that are based only on non-intrusive sensor data.

**Methods:**

A wireless sensor system with ten sensor boxes was installed in the home of ten healthy subjects to collect ambient data over a duration of 20 consecutive days. A handheld protocol device and a paper logbook were also provided to the subjects. Eight ADL were selected for recognition. We developed two ad-hoc ADL classifiers, namely the rule based forward chaining inference engine (RBI) classifier and the circadian activity rhythm (CAR) classifier. The RBI classifier finds facts in data and matches them against the rules. The CAR classifier works within a framework to automatically rate routine activities to detect regular repeating patterns of behavior. For comparison, two state-of-the-art [Naïves Bayes (NB), Random Forest (RF)] classifiers have also been used. All classifiers were validated with the collected data sets for classification and recognition of the eight specific ADL.

**Results:**

Out of a total of 1,373 ADL, the RBI classifier correctly determined 1,264, while missing 109 and the CAR determined 1,305 while missing 68 ADL. The RBI and CAR classifier recognized activities with an average sensitivity of 91.27 and 94.36%, respectively, outperforming both RF and NB.

**Conclusions:**

The performance of the classifiers varied significantly and shows that the classifier plays an important role in ADL recognition. Both RBI and CAR classifier performed better than existing state-of-the-art (NB, RF) on all ADL. Of the two ad-hoc classifiers, the CAR classifier was more accurate and is likely to be better suited than the RBI for distinguishing and recognizing complex ADL.

**Electronic supplementary material:**

The online version of this article (doi:10.1186/s12938-015-0050-4) contains supplementary material, which is available to authorized users.

## Background

Activities of daily living (ADL) is an umbrella term that refers to self-care, comprising activities or tasks that people undertake routinely in their everyday life. ADL are the essential activities a person needs to perform to be able to live independently. Existing literature identified the importance of ADL such as bathing, toileting, eating [1] as indicators of the physical and cognitive abilities of elderly individuals. ADL have been shown to predict the functional capacity in healthy adults and elderly people [1] and admission to a nursing home [2].

Activities of daily living assessment measures individuals’ ability to formulate and plan goals in interaction with the environment in which they live and are routinely used by physicians/clinicians. Decline in ADL performance was shown to reflect cognitive impairment, neurobehavioral dysfunction or other neurological disorder/injury [3]. Measuring impairments in ADL performance is important, but difficult to be assessed and recognized outside the native environment of a person (e.g. doctor’s office). Moreover, it is an important information for professional caregivers to optimize medication and to personalize care [4].

Activities of daily living are traditionally assessed with questionnaires like the Katz Index of Independence in Activities of Daily Living (Katz ADL) [1], Stanford Health Assessment Questionnaire [5] and the Barthel ADL Index [6]. The Katz ADL [1] ranks adequacy of performance in the six functions of bathing, dressing, toileting, transferring, continence and feeding. Questionnaire based ADL assessments are challenging as they rely on informant information. In addition, self-reported data are subject to bias and errors due to cognitive impairments or lack of insight.

The automatic assessment of ADL is a fundamental problem in elderly care. Several groups have proposed sensor-based systems to recognize and quantify ADL [7–11] in the patient’s home. In so-called smart homes, several sensors, e.g. accelerometers, microphone arrays, pressure sensitive mats, gas sensors and cameras are installed in the proximity of older patients to determine specific activities and to monitor their ability of coping with ADL [8, 9]. Other types of methods used to detect and recognise activities within a home include wearable sensors [12, 13], as well as data received from a residential power line [14, 15]. Wearable sensors are usually used to gather physiological and movement data [12], while cameras are used to detect a variety of activities, including sign language recognition, human gait recognition, sitting, standing and walking behaviors.

Irrespective of the sensor-type, all sensor systems require processing and classification of the massive amount of collected data to derive information regarding the ADL. Different algorithms have been used to classify and recognize ADL from sensor streams, such as probabilistic based [16], rule based [15], Gaussian Mixture Models [7], K-means clustering [15], Support Vector Machine (SVM) [17], Hidden Markov Models (HMM) [13, 18] and the Naïve Bayes (NB) classifier [18, 19]. The majority of these algorithms are training-based. Despite being quite simple, NB often delivers good results making it a good state-of-the art algorithm for first data assessments. Furthermore, the performance of NB gives better insight into the “complexity” of the underlying patterns than SVM or HMM. SVM and HMM are already designed to work with complicated data. The Random Forest (RF) [20] belongs to the new generation of classification schemes and has been used in clinical applications [21].

This paper describes the two ad-hoc classifier algorithms (rule based, pattern based) we developed and evaluates them using wireless sensor data collected from the homes of ten healthy subjects. The performance of the ad-hoc classifiers is also validated using the NB and RF classifiers. The choice of the state-of-the-art classifiers was based on common practice of first data assessment with the NB, and on novelty and resistance to over-fitting performance, shown by many data mining and machine learning researchers, for RF [20]. Firstly, we introduce the rule based forward chaining inference engine (RBI) classifier that finds facts in data and matches them against rules until a conclusion is reached. Inferencing in the context of rule-based system means to process the supplied data and the stored knowledge, in order to produce correct conclusions. A rule based inference engine using forward chaining searches the inference rules until it finds one where the rules match. When such a rule is found, the engine can conclude or infer the new fact to be added to the rule database. Forward chaining starts with the available data and uses inference rules to extract more data until a goal is reached.

Secondly, we introduce the circadian activity rhythm (CAR) classifier, which works within a framework to automatically rate routine activities and detect regular patterns of behavior. The human body keeps track of time in a section of the brain called the suprachiasmatic nucleus [22]. Clinical observations have shown that human functions follow periodical variations regulated by internal biological rhythms. When the period of the cycle approximates 24 h, it is qualified as circadian. Hunger, for instance, represents a circadian rhythm that is regulated by hormones and activity. Our hypothesis was that measuring the circadian variability of the ADL could improve the recognition accuracy of complex ADL.

## Methods

### Sensor system

**Figure 1:**
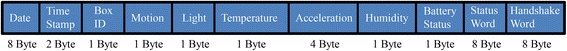
Data packet. The contents of the data packet transmitted by the sensor boxes to the central computing unit. The Box ID contains the room information while the 4 bytes of the acceleration represent the node acceleration, acceleration in x-axis, y-axis and z-axis.

The wireless sensor system [23] comprises of ten sensors boxes and a central computing unit (CCU). Five sensors which capture ambient values [i.e. temperature (°C) (DS18B20, Dallas Inc.), humidity (g/m^3^) (SHT21P, SENSIRION), luminescence (l×) (AMS302, Panasonic Inc.), passive infrared radiation (V) (EKMB1101111, Panasonic Inc.) and acceleration (m/s^2^) (ADXL345, Analog Device)] are assembled within one sensor box (*l* × *w* × *h* = 15 mm × 30 mm × 60 mm, weight = 80 g). A commercially available laptop, running customized Microsoft Windows 7 software, serves as the CCU and acts as a local data server. The ad-hoc classifiers implemented in Matlab R2007b (The MathWorks, Inc.) and state-of-the-art classifier implemented in KNIME (https://www.knime.org/knime.org) run on the CCU too. A receiver unit attached to the CCU collects the data packets transmitted from the ten sensor boxes. Besides the five ambient sensor values, each data packet includes a date, timestamp, sensor node number, supply voltage, status word with even-parity error handling and a handshake word to detect frame collision as shown in Figure 1. A wireless protocol device [23] built in a housing with a wearable belt clip and fitted with switches corresponding to the selected ADL serves as an electronic logbook.

### Subject recruitment

The data collection was carried out in accordance with the latest version of the Declaration of Helsinki and was approved by the Ethics Committee of the Canton of Bern, Switzerland. All procedures related to the study were explained to the participants and a written informed consent was obtained prior to participation. No compensation for participation was provided. Healthy subjects were recruited via advertisement in the local newspaper and in the Senior University of Bern. Demographic details were collected using standard questionnaires. Subjects were assessed for neuropsychological details with standardized paper–pencil test battery, which included the Montreal Cognitive Assessment (MoCA) [24, 25], the Trail Making Test A and B (TMT-A, TMT-B) [26] and the timed “Up & Go” test [27, 28]. Subjects with cognitive impairment (MoCA score ≤26), or significant motor impairment (timed “Up & Go” ≥12 s) or sharing the house with others or not living alone were excluded. Ten healthy subjects (6 women, 4 men; age 28–79 years) were included in the study. The data were collected continuously for 20 consecutive days per subject.

### ADL selection

Activities of daily living such as sleeping, grooming, toileting, getting ready for bed, cooking, eating, watching TV and seated activity were chosen for this study. The eight ADL (Table 1) and their definition were used to define the parameters required for classification.

**Table 1:** Definition of the eight ADL selected for recognition

ADL	Included activities	Excluded activities
Sleeping	Resting at night, a nap either in bed on couch	Lying down for recovery
Grooming	Personal hygiene	Simple toileting and hand washing
Toileting	Simple toileting and washing hands	Other or additional personal hygiene
Getting ready for bed	Personal hygiene before bedtime	Pre-bedtime rituals
Cooking	Preparing food in the kitchen	Cutting pizza from delivery service, making popcorn, etc., making tea or coffee
Eating	Having a meal (also delivered food)	Snack or having just a cup of coffee or a glass of water
Watching TV	Watching TV with main focus on the TV	Other activities while the TV is just on
Seated activity	Sitting at a table or in an easy chair	Taking a nap in a chair

*ADL* activity of daily living.

### System setup

The sensor boxes and the CCU were installed in the home of the ten healthy subjects. For each room, one sensor box was fixed at a height of approximately 2 m, facing towards the middle of the room. Additional sensor boxes were placed in the kitchen (on the fridge door) and in the bathroom (on the flush handle). Once the sensor system was set up and initialized, it recorded the five ambient environmental values autonomously at a rate of 0.2 Hz and continuously for a duration of 20 days in the participant’s home. For validation, the subjects were instructed to protocol their ADL using the wireless protocol device and a paper–pencil log book.

### Data handling

Prior to classification, the collected data were stratified by rooms in the apartment [23] using a Bucketsort algorithm [29]. The sensor node number recorded during data acquisition provides the room information. The data for each room were then sorted in a chronological order by using a Radixsort algorithm [30].

### Data analysis: ADL classification

Data analysis was based both on existing classification (NB and RF) algorithms and two ad-hoc (RBI and CAR), in-house developed algorithms. The goal of classification is to assign the sorted ambient sensor values to a given set of ADL. The supervised training approach based classifiers such as the NB and RF use a training-set data to predict the classification. Our ad-hoc classification algorithm is based on the assumption that irrespective of the daily routine of the subject, specific patterns with specific duration and timing occur every day [31].

### Naïve Bayes (NB) classifier

This classifier is based on Bayes theorem [32], which assumes that the features are independent. Bayes conditional probability model is then combined with a decision rule, which picks the most probable hypothesis. This is done by maximising the posterior probability, and thereby assigning a class-label to a given input vector.

### Random Forest (RF) classifier

The RF [33] belongs to one of the newest algorithm and is a non-probabilistic decision-tree based classifier. The algorithm generates a number of decision-trees. For each tree, only a random subset of the available data is considered. Additionally at each node, only a random subset of all features is used for the split. No pruning is performed on the final trees. To classify new data, it is feed into each tree, so that a majority vote over all trees decides which class-label is assigned. The advantages of the RF classifier is its resistance to overfitting which guarantees generalisation to new data.

### Rule based inference (RBI) classifier

**Figure 2:**
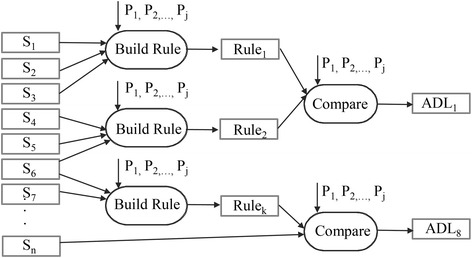
Forward chaining inference engine. *Block* *diagram* showing the forward chaining inference engine of the rule based inference (RBI) classifier. The forward chaining engine receives the sorted data from the database of facts and behavioural parameters (*P*_*1*_, *P*_*2*_, *P*_*3*_, *P*_*j*_) the rule repository. The sorted data from the database of facts are converted to ambient value matrices (*S*_*i*_). The rules (*Rule*_*1*_, *Rule*_*2*_, *Rule*_*3*_,…, *Rule*_*k*_) are built upfront using the behavioural parameters.

The RBI classifier is built on (1) a database of facts, (2) a rule-repository and (3) a forward chaining inference engine [23]. The database of facts is a collection of data sorted by the Bucketsort [29] and Radixsort [30] algorithms, but also previously classified ADL data (historical). The rule-repository provides the forward chaining inference engine with a set of parameterized behavioral knowledge $(P_{1} ,P_{2} , \ldots ,P_{j}$). The behavioral knowledge was defined by our medical team which consists of parameters such as minimum duration required for classification of the ADL sleeping or time defined for routine meal activities. A parser translates the parameterized behavioral knowledge into a lookup table disposable in the Random-Access Memory of the CCU. The forward chaining inference engine (Figure 2) then checks if the available data fulfills the defined rules and behaviors to be classified as a specific ADL and runs using three basic components:

1. $$Ambient\,value\,matrices = S_{{1_{{t_{end} - t_{0} }} }} ,S_{{2_{{t_{end} - t_{0} }} }} , \ldots ,S_{{i_{{t_{end} - t_{0} }} }}$$
2. $$Behavioural\,parameters = \left( {P_{1} ,P_{2} , \ldots ,P_{j} } \right)$$
3. $$Rules = \left( {Rule_{1} ,Rule_{2} , \ldots ,Rule_{k} } \right) .$$

For each potential ADL, an ambient value matrix (*S*_*i*_) is composed from the sorted raw data. Each ambient value matrix, *S*_*i*_ is then compared to a set of rules $\left( {Rule_{1} ,Rule_{2} , \ldots ,Rule_{k} } \right)$ defined upfront for each of the eight ADL. The rules are defined manually and are the same for all subjects. For example, the values of the humidity sensor must increase to detect the action of grooming in the bathroom. The rules are built under the premise of the behavioral parameters. For example, cooking can only take place in the kitchen. Data not captured in the kitchen can be neglected when it comes to the determination of the ADL cooking. The rule “cooking can only take place in the kitchen” is a must and hence a very top level rule for an ad-hoc classifier. On the other end there are very low level rules like “switching of lights when leaving the kitchen”. After cooking, switching of the lights is the most likely case, but it is not necessary. Hence, “switching of lights when leaving kitchen” is likely, but not a requirement for the ADL cooking.

$$\begin{array}{c} Rule_{1} = (S_{{1_{{t_{end} - t_{0} }} }} >P_{1} )\bigwedge(S_{{2_{{t_{end} - t_{0} }} }} = P_{2})\bigwedge \cdots \bigwedge(S_{{i_{{t_{end} - t_{0} }} }} \ge P_{j} ) \\Rule_{2} = (S_{{3_{{t_{end} - t_{0} }} }} \le P_{3})\bigwedge(S_{{1_{{t_{end} - t_{0} }} }} > P_{4} )\bigwedge\cdots \bigwedge (S_{{i_{{t_{end} -t_{0} }} }} = P_{j} ) \\ \cdot \\ \cdot \\\cdot \\ Rule_{k} = (S_{{2_{{t_{end} - t_{0} }} }} =P_{4} )\bigwedge(S_{{4_{{t_{end} - t_{0} }} }} \le P_{2})\bigwedge \cdots \bigwedge(S_{{i_{{t_{end} - t_{0} }} }} \ne P_{j} ) \\\end{array}$$

A conflict resolution strategy is implemented within the forward chaining inference engine to decide the order of information processing. Depending on the complexity of the ADL classified, n-steps of iteration were used. By processing the daily routine with the RBI classifier, the system allots specific patterns to one of the eight ADL. The output of the RBI classifier is the recognized ADL a subject performed at a given time on a day.

### Circadian activities rhythm (CAR) classifier

**Figure 3:**
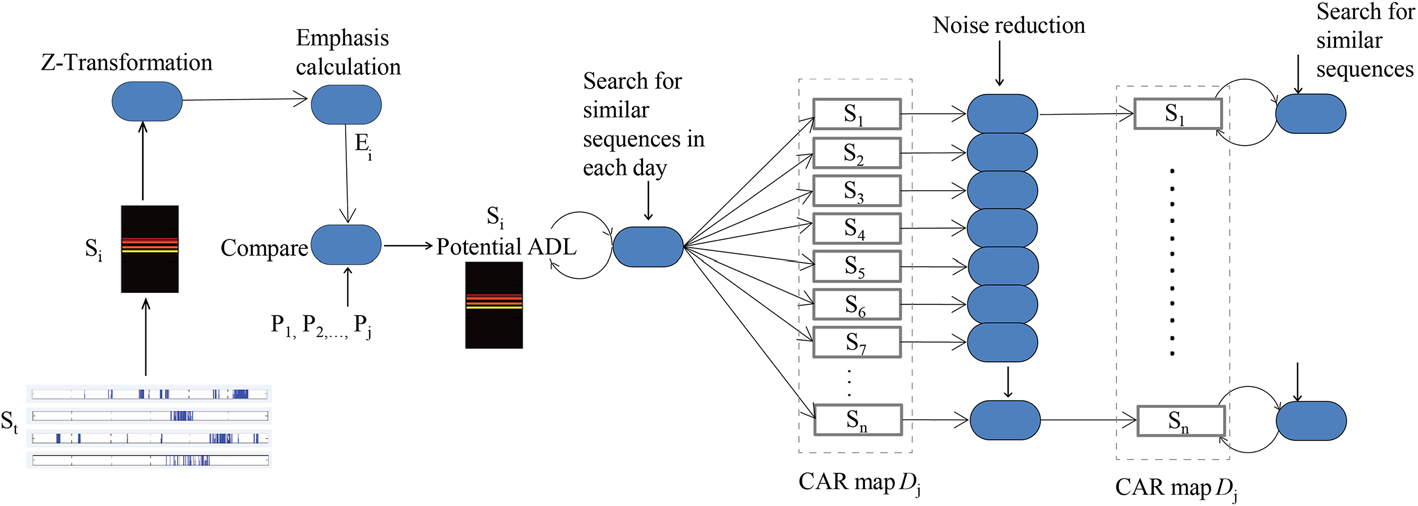
CAR classifier schematic. Schematic of data processing sequences in a circadian activity rhythm (CAR) classifier. The sorted data is composed into ambient value matrices (*S*_*i*_) and transformed using z-transformation. The emphasis of the matrices *S*_*i*_ are calculated before detecting regular patterns in each of them.

Biological circadian rhythms are mainly based on daylight and are characterized by their amplitude, period and phase [22]. The daily activities of humans also periodically fluctuate and are dependent on the circadian rhythms. The CAR classifier is based on measuring the circadian variability of an activity by recognizing rhythmic patterns with small fluctuations for an activity. It is built on the idea of pattern recognition with a core algorithm, which analyses sequences [34] of ambient value matrices (*S*_i_). Figure 3 shows the sequence of data processing and analysis in the CAR classifier.

After sortation, the ambient values samples (*S*_t_) are composed into ambient value matrices (*S*_i_). This is done by summing up the *S*_t_ chronologically as long as the next sample is not null.

$$S_{i} = \mathop \sum \limits_{{t_{start} }}^{{t_{end} }} s_{t} | s_{{\left( {t + 1} \right)}} \ne 0$$

As each subjects performs or behaves differently in his or her home environment, there is inter-subject variability leading to unknown parameters for *S*_i_. For further computation, the *S*_i_ are standardized by z-Transformation:

$$S_{i} = \left| {\frac{{S_{i} - \mathop {S_{i} }\limits^{ - } }}{SD}} \right|$$

Next, the emphasis of the *S*_i_ are calculated to detect data sequences with unique or pronounced patterns.

$$E_{i} \left( {S_{i} ,s_{t} } \right) = \frac{1}{{S_{i} }}*\mathop \sum \nolimits t*s_{t}$$

The obtained emphasis *E*_*i*_ is then compared to a set of parameterized behavioural knowledge $\left( {P_{ 1} ,P_{ 2} , \ldots ,P_{\text{j}} } \right)$ defined by our medical team. If the emphasis *E*_i_ of a *S*_i_ matches the parameters of the behavioural knowledge base, the *S*_i_ is stated as a potential ADL. Based on this information, similar *S*_i_ or *S*_i_ sequences can be found throughout each day. By assembling these similar *S*_i_ sequences according to their apportionment a CAR map *D*_*j*_ is obtained:

$$D_{j} = \left\{ {S_{1} ;S_{2} ;S_{3} ; \ldots ;S_{i} } \right\}$$

In order to reduce noise and eliminate out layers, brought in by sensor fluctuations and transmission errors, a Gaussian broad band filter is applied:

$$S_{i} \left( h \right) = \frac{1}{{SD^{2} *\sqrt {2\pi } }}*e^{{\frac{{ - i^{2} }}{{2SD^{4} }}}}$$

**Figure 4:**
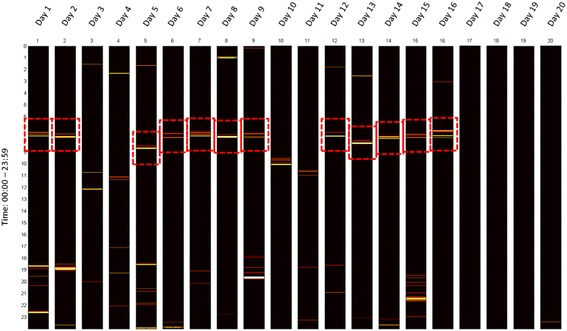
Regular patterns: CAR maps. The CAR classifier recognises similar patterns throughout each day in the ambient value matrices (*S*_*i*_) and checks for the time of occurrence, duration, and duration w.r.t to other sequences/patterns to recognise the ADL.

In a final step, similar sequences throughout each day (Figure 4) are analysed and determined. By analysing the occurrence and duration of each sequence in relation to other sequences, one of the eight ADL is allotted.

### Threshold to remove false positives

To remove false positives, we implemented a simple thresholding approach which removed all predicted activities that lasted less than 20 s.

### Classification performance

To evaluate the activity recognition performance of the NB and RF classifiers, a leave-one-out [35] cross-validation was performed on the ADL data. Leave-one-out is a special case of the k-folds validation. During each step of cross-validation, we trained our system with the data of nine subjects. We then used the trained system on the remaining (tenth) subject, to label the ADL. This cross-validation of training and testing was performed for all of the ten possible combinations.

**Figure 5:**
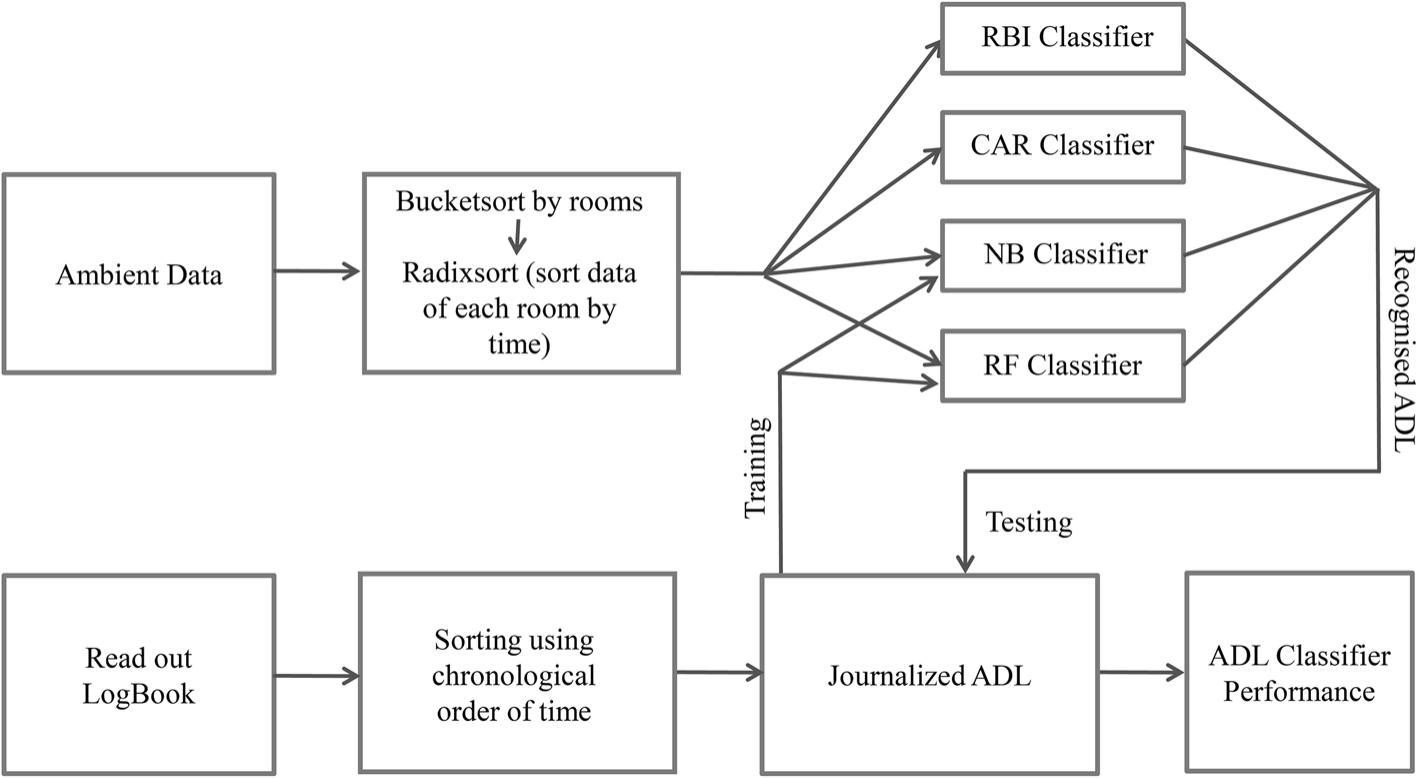
Validation schematic. Cross-validation of ADL classifier output with the wireless protocol box data/paper–pencil log book. The sensitivity and specificity of the classifiers are calculated using the logged data as the ground truth.

The activity log from the wireless protocol device and the paper–pencil log book were merged to a single journalized ADL log which served as the ground truth for comparison with the classifier’s output (Figure 5). The performances (sensitivity and specificity) were calculated by cross-validating the output of the four classifiers with the journalized ADL log.

### Activity maps

The activity map is a visualization technique which makes it possible to analyse the complete whole data at once. It is both a visual technique which can make use of the human eye’s ability to recognize patterns, and a quantitative one, in the sense that it introduces coloured sequences which quantify the information contained in the data patterns. For the RBI and CAR classifier, the recognized ADL for each subject were plotted against the time period of 20 days to generate an activity map.

## Results

### Demographics

The demographics of the healthy subjects are summarized in Table 2. The MoCA score, TMT-A, TMT-B and timed “Up & Go” scores were in a normal, non-pathological range.

**Table 2:** Demographics and clinical parameters of the healthy subjects

Demographics	Healthy subjects, *n* = 10
Age (years) (mean ± SD [range])	48.8 ± 20.0 [28–79]
Gender (m/f) (% male)	4/6 (40)
MoCA score (max = 30) (mean ± SD [range])	29.1 ± 1.14 [28–30]
Timed Up & Go (s) (mean ± SD [range])	8.2 ± 1.3 [7.0–9.0]
Trial Making Test A (s) (mean ± SD [range])	39.1 ± 20.0 [15–66]
Trial Making Test B (s) (mean ± SD [range])	62.6 ± 32.3 [21–97]

*MoCA* Montreal Cognitive Assessment.

### Data acquisition and transmission

The wireless sensors distributed in the home of the ten subjects (20 days/subject) successfully captured 33,939,441 data packets (543,031,056 environmental values). A small percentage (0.47%, 160,269 data packets) of the captured data packets were lost due to transmission errors, hence leading to an overall transmission reliability of 99.53%.

### ADL classification and performance comparison

For the ad-hoc classifiers, the sorting and analysis of the captured data packets retrieved a total of 1,373 ADL. The RBI classifier correctly determined 1,264 ADL and missed 109 ADL, while the CAR classifier determined 1,305 ADL and missed 68 ADL. Additional file 1: Table S1 shows the number of missed and correctly classified ADL by the different classifiers. The state-of-the-art algorithms retrieved a total of 18,214 ADL datasets, whereof 5,032 were correctly recognised by NB and 13,971 by RF classifiers.

The difference in sensitivity and specificity for the individual ADL between the classifiers are shown in Table 3. The RF classifier with an average specificity of 97.07% and sensitivity of 60.36% performed better than the NB classifier whose mean specificity was 90.61%. The CAR classifier achieved high sensitivity for grooming (97.78%) and toileting (96.09%). Cooking proved to be the most challenging activity for the RBI classifier while both cooking and eating were challenging for the CAR. The CAR classifier performed better than the RBI (sensitivity 91.27%, specificity 92.52%) [23] with a result of 94.36% for sensitivity and 98.17% for specificity.

**Table 3:** Performance of state-of the-art and ad-hoc classifiers

Activities of daily living	State-of the-art classifiers				Ad hoc classifiers			
	Naive Bayes		Random Forest		Rule based inference		Circadian activity rhythm	
	Sens.	Spec.	Sens.	Spec.	Sens.	Spec.	Sens.	Spec.
Sleeping	91.85	40.99	98.08	84.71	93.64	85.77	95.95	95.58
Grooming	27.03	96.67	55.58	99.62	94.07	96.98	97.78	98.91
Toileting	23.95	96.92	52.53	99.61	94.79	91.54	96.09	98.23
Getting ready for bed	9.53	98.52	43.03	99.86	92.38	94.48	95.24	98.39
Cooking	26.88	98.83	47.28	99.92	84.29	90.92	91.43	98.23
Eating	1.34	99.40	47.35	99.97	87.78	94.83	90.00	98.85
Watching TV	18.41	95.31	85.36	93.81	93.17	90.63	95.96	98.28
Seated activity	12.90	98.25	53.67	99.05	90.06	94.98	92.40	98.92
Mean	26.49	90.61	60.36	97.07	91.27	92.52	94.36	98.17

All values are represented as %.

*Sens.* sensitivity, *Spec.* specificity.

**Figure 6:**
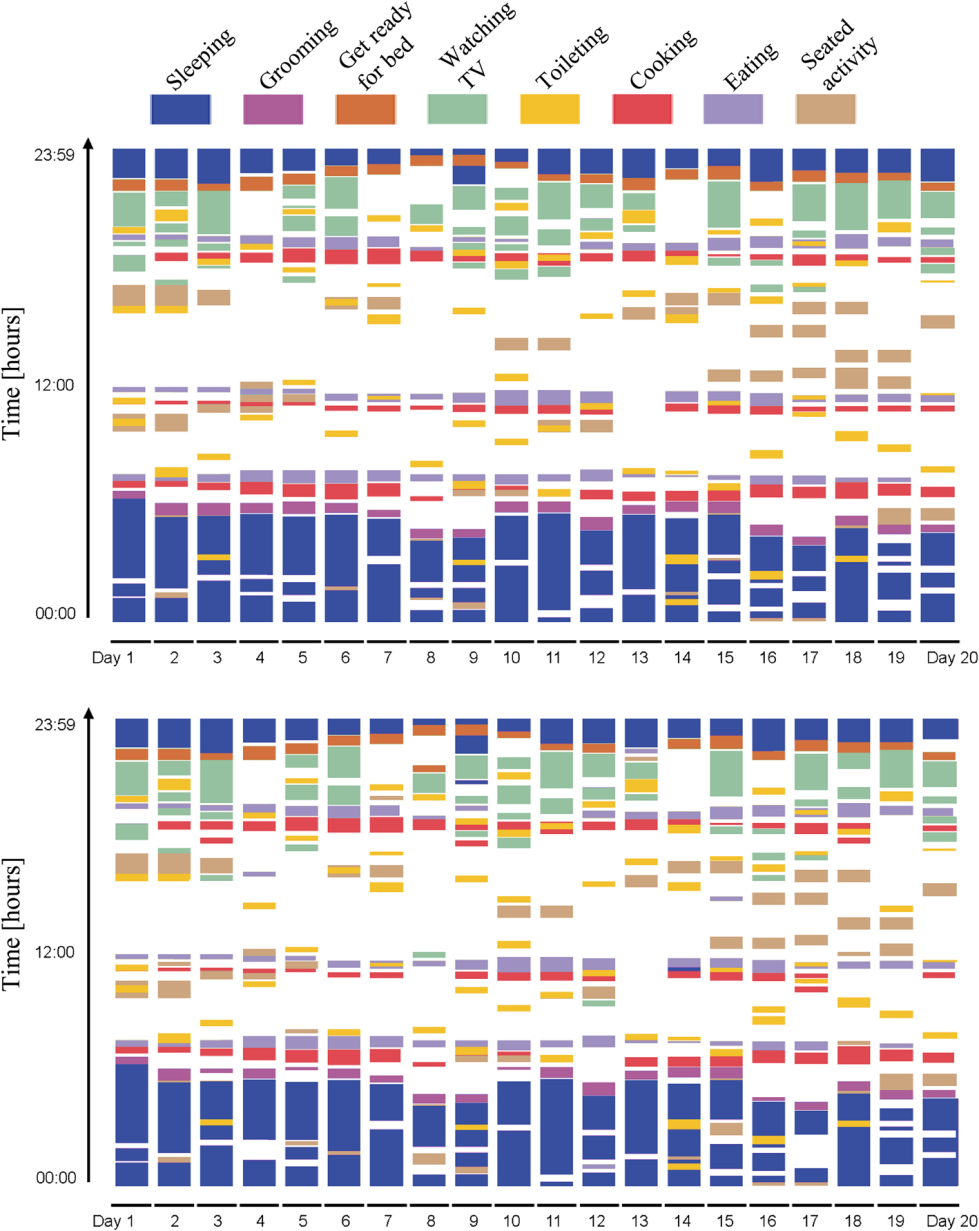
Activity maps. Activity maps are qualitative means to visualize the recognized ADL. This activity map visualizes the ADL recognized from sensor data of a healthy female subject (age 75, Montreal Cognitive Assessment score 29) using CAR (*above*) in comparison with the RBI [23] (*below*) classifier.

The activity map of a healthy female subject (age 75, MoCA 29) shown in Figure 6, displays the correctly recognised ADL from the data collected over 20 days using our ad-hoc classifiers. There are subtle differences in the visualised activity map of the same subject using the RBI and CAR classifiers.

## Discussion

Ambient assisted living applications are on the rise [36] and hence evaluation of classifiers are important for their deployment. In addition, validation of classifiers should be considered as part of establishing the clinical validity of the recognition technique [37]. We evaluated the NB, RF, RBI and CAR classifiers and demonstrated that all classifiers recognize ADL from data collected via the wireless sensor system. The comparison between the classifiers indicated that the best classification results were achieved using the CAR classifier. The CAR classifier does not require the training data, which makes it promising for easy and fast deployment. A good classifier is a vital parameter for a sensor system [38] and thus selecting the right classifier is important.

Some ADL are not exclusively room related and evoke only slightly different ambient values as reported earlier [16, 17, 39]. In our study, the spatial information is obtained from the sensor node number which recognizes the room of ADL. Spatial information helps increasing the accuracy of classification which is justified with our results. Highest accuracies were achieved for room specific ADL such as grooming with RBI and CAR and for toileting, cooking, eating with NB and RF. However, our algorithms have not taken the temporal information (limiting activity to time of the day) into consideration. This is an advantage, useful to extend our application to different kinds of patients and applications.

Specificity and accuracy of ADL recognition have been reported before and our results are in line with them [16, 17]. The SVM models used to recognise specific ADL using data captured from video cameras and microphones, wearable kinetic sensors achieved a sensitivity of 97.80% [17]. Single and combined classifiers have been compared before for specificity and sensitivity of ADL detection and shown that combined classifiers outperform single classifiers [40]. Specificity (99.6%) for routine ADL have been reported using a waist-worn wireless tri-axial accelerometer with an ensemble of classifiers [40]. The CAR classifier results are in line with these results, while the RBI classifier performance falls below this level.

Accurate models of ADL require labelled examples of ADL for training, however comparable ADL inevitably vary between households and between individuals. The protocol box and log book provided ADL labels for cross-validation and were used for training in the NB and RF classifiers, though not for training for the ad-hoc classifiers. However, we should not rule out the possibility of subjects reporting a wrong activity or missing an activity. This compliance in logging ADL negatively affects the performance of ADL recognition and may be one of the reason that performance for some ADL are low.

The majority of the algorithms reported earlier are usually embedded in other algorithms and make up only part of the final results, which makes a comparison of the different algorithms difficult. It is possible that one classifier can obtain very high accuracy for one ADL and average accuracy for another ADL, while the other classifier obtains moderate accuracy for both ADL. When such a condition arises it is difficult to predict the best classifier. In our case, the CAR classifier performed better than RBI for all ADL which makes the comparison easier. The RBI classifier can handle unusual data by adding another rule. But with the manual implementation of a rule-based algorithm there is a risk for over fitting and the performance on new data is non-predictable. The CAR classifier uses the fact that a person’s behavior during the daily cycle tends to fall into regular patterns and variation in these circadian patterns can be pre-markers of upcoming diseases. CAR based approaches have great potential in monitoring activity using ambient sensor systems and are expected to make “aging in place” a possibility for elderly people and patients with dementia [10, 11].

Activity maps can provide information about the variations or transitions in ADL patterns. This may help to benchmark the physical and cognitive abilities of patients [1]. The difference in activity maps between the two ad-hoc classifiers are may be too small and can be ignored by the human eye, however, changing the threshold window size the small differences between patients can be tuned in the activity map. An activity like sleeping, characterized by longer duration, can be best viewed with the activity maps. In addition, using threshold window size to exclude activities of very short duration improves classification performance.

The advantages of our wireless sensor system lie in its low maintenance, quick and easy installation, small discrete shape, non-intrusiveness into privacy of subjects and model-independent data collection. The sensor system further does not draw attention from the user and can be used discreetly. The classification performance in our study relied on the protocol compliance (wireless protocol box and paper–pencil log book) of the subjects. One possible downfall of this approach is that subjects might have forgotten to protocol an activity or logged a wrong ADL, which in turn may have affected the classification performance. While using the log book approach is far from flawless, it seems that the classifier performances reported in this paper actually represent a worst case scenario. Moreover, the ADL recognized and reported here are activities performed by subjects in a free-living context, where subjects made natural transitions between activities. We have classified a wide range of activities ranging from static “sleeping” to the more dynamic “cooking” compared to most studies which limit themselves to two or three ADL. The accuracies obtained with the wide range of ADL in our study is promising. State-of-the-art classifiers as well as custom built algorithms can be used with our wireless sensor system. As for limitations, we collected data using a small sample of participants. Future studies would benefit from using multiple demographic participant designs, as daily activity patterns vary with individuals.

## Conclusion

The non-intrusive wireless sensor system can be used to acquire environmental data essential for the classification of ADL. Both the ad-hoc classifiers performed better than the state-of-art classifiers. The pattern recognition based CAR classifier performed better than the RBI and we surmise that it is better suited for complex ADL. Variations in regular circadian patterns are thus measurable by monitoring activity and hold great promise in early detection of disorders.

## Additional files

**Additional file 1: Table S1** Confusion matrix for the classification of data.

## Abbreviations

ADL: activities of daily living
RBI: rule based forward chaining inference engine
CAR: circadian activity rhythm
NB: Naïve Bayes
RF: Random Forest
SVM: Support Vector Machine
HMM: Hidden Markov Models
