# Supplementary material for: Recognition of activities of daily living in healthy subjects using two ad-hoc classifiers
Source: Biomed Eng Online. 2015 Jun 6;14:54. doi: 10.1186/s12938-015-0050-4 (PMC4457983; doi:10.1186/s12938-015-0050-4)
Supplement: Supplementary file 1 — Additional file 1: Table S1 Confusion matrix for the classification of data. [file 12938_2015_50_MOESM1_ESM.doc]

**Table 3 - Confusion matrix for the classification of data**

| **Activities of daily living** | **Naïve Bayes**  **NB**  **NB** | | | | **Random Forest** | | | | **Rule based inference** | | | | **Circadian activity rhythm** | | | |
| --- | --- | --- | --- | --- | --- | --- | --- | --- | --- | --- | --- | --- | --- | --- | --- | --- |
|  | **FP** | **FN** | **TN** | **TP** | **FP** | **FN** | **TN** | **TP** | **FP** | **FN** | **TN** | **TP** | **FP** | **FN** | **TN** | **TP** |
| Sleeping | 3.32 | 4.77 | 90.65 | 1.26 | 1.28 | 1.21 | 92.69 | 4.82 | 10.60 | 1.77 | 63.92 | 23.71 | 3.38 | 0.95 | 73.21 | 22.46 |
| Grooming | 0.98 | 21.51 | 69.15 | 8.36 | 3.61 | 1.39 | 66.52 | 28.49 | 2.81 | 0.42 | 90.17 | 6.61 | 0.95 | 0.16 | 91.90 | 6.98 |
| Toileting | 34.59 | 5.00 | 55.43 | 4.98 | 6.59 | 1.78 | 83.42 | 8.20 | 6.89 | 0.97 | 74.55 | 17.59 | 1.42 | 0.77 | 78.76 | 19.04 |
| Getting ready for bed | 18.68 | 2.78 | 68.53 | 10.00 | 1.05 | 3.94 | 86.16 | 8.85 | 5.21 | 0.43 | 89.15 | 5.21 | 1.51 | 0.29 | 92.40 | 5.80 |
| Cooking | 7.18 | 4.95 | 86.21 | 1.65 | 2.33 | 2.72 | 91.06 | 3.89 | 8.72 | 0.61 | 87.37 | 3.29 | 1.70 | 0.35 | 94.20 | 3.75 |
| Eating | 4.90 | 3.61 | 91.28 | 0.21 | 0.40 | 2.19 | 95.78 | 1.64 | 5.05 | 0.31 | 92.60 | 2.04 | 1.11 | 0.29 | 96.20 | 2.58 |
| Watching TV | 2.21 | 19.06 | 77.65 | 1.08 | 6.12 | 3.05 | 73.74 | 17.09 | 7.80 | 1.14 | 75.46 | 15.60 | 1.42 | 0.71 | 81.06 | 16.82 |
| Seated activity | 0.52 | 10.68 | 88.72 | 0.08 | 1.91 | 7.02 | 87.33 | 3.74 | 4.80 | 0.52 | 90.77 | 3.92 | 1.02 | 0.46 | 92.98 | 5.55 |
| TOTAL | 9.05 | 9.05 | 78.45 | 3.45 | 2.91 | 2.91 | 84.59 | 9.59 | 5.83 | 0.62 | 86.33 | 7.22 | 1.36 | 0.44 | 89.74 | 8.46 |

FP: False positive; FN: False negative; TN: True negative; TP: True negative; All values are represented as %
